# Aflatoxin B_1_ affects apoptosis and expression of death receptor and endoplasmic reticulum molecules in chicken spleen

**DOI:** 10.18632/oncotarget.20595

**Published:** 2017-08-30

**Authors:** Panpan Zhu, Zhicai Zuo, Zhixiang Zheng, Fengyuan Wang, Xi Peng, Jing Fang, Hengmin Cui, Caixia Gao, Hetao Song, Yi Zhou, Xici Liu

**Affiliations:** ^1^ Key Laboratory of Animal Diseases and Environmental Hazards of Sichuan Province, College of Veterinary Medicine, Sichuan Agricultural University, Chengdu, Sichuan 611130, PR China; ^2^ College of Veterinary Medicine, Sichuan Agricultural University, Chengdu, Sichuan 611130, PR China; ^3^ College of Life Sciences, China West Normal University, Nanchong, Sichuan 637002, PR China; ^4^ Life Science Department, Sichuan Agricultural University, Yaan, Sichuan 625014, PR China

**Keywords:** AFB_1_, splenocyte apoptosis, death receptor molecules, endoplasmic reticulum molecules, chicken

## Abstract

Aflatoxin B_1_ (AFB_1_) is a natural product of the Aspergillus genus of molds, which grow on several foodstuffs stored in hot moist conditions, and is among the most potent hepatocarcinogens and immunosuppression presently known. The latter was related to the up-regulated apoptosis of immune organs. However, the effect of expression of death receptor and endoplasmic reticulum molecules in AFB_1_-induced apoptosis of chicken splenocytes was largely unknown. The objective of this study was to investigate this unknown field. One hundred and forty four one-day-old chickens were randomly divided into control group (0 mg/kg AFB_1_) and AFB_1_ group (0.6 mg/kg AFB_1_), respectively and fed with AFB_1_ for 21 days. Histological observation demonstrated that AFB_1_ caused slight congestion and lymphocytic depletion in the spleen. TUNEL and flow cytometry assays showed the excessive apoptosis of splenocytes provoked by AFB_1_. Moreover, quantitative real-time PCR analysis revealed that AFB_1_ induced the elevated mRNA expression of Fas, FasL, TNF-α, TNF-R_1_, Caspase-3, Caspase-8, Caspase-10, Grp78 and Grp94 in the spleen. These findings suggested that AFB_1_ could lead the excessive apoptosis and alter the expression of death receptor and endoplasmic reticulum molecules in chicken spleen.

## INTRODUCTION

Aflatoxins were difuranocoumarin compounds, and included B_1_, B_2_, G_1_, G_2_, M_1_, and M_2_, among which aflatoxin B_1_ (AFB_1_) showed highly hepatotoxic, genotoxic, immunotoxic and other adverse effects on humans and animals [1–7].

Secondary to the effect on liver, the immunosuppressive nature of AFB_1_ is the best documented area of its toxicity [4]. AFB_1_ inhibited the development of bursa of Fabricius [8], thymus [9], affected the weight and function of immune organs [10], decreased the percentages of T cell subsets, reduced the Ig-containing cell number [11] and the counts of splenic plasma cells [12], depressed the mitosis of B cells [13] and immunoglobulin as well as antibody production [14, 15]. In addition, AFB_1_ caused oxidative stress in lymphoid tissue [16, 17], cell cycle arrest [18], and mitochondria injury in the lymphoid organs [19].

Apoptosis is associated with the normal development and homeostasis of animal tissues, and also involves in the pathogenesis [20]. Available information revealed that AFB_1_ caused excessive apoptosis of several poultry and mammal cells such as hepatocytes [21], thymocytes [19, 22], splenocytes [17], bursa of Fabricius cells [8], bronchial epithelial cells [23], jejunal mucosal cells [24], bone marrow cells [25], and renal cells [26]. Furthermore, our early researches have shown the possible link between mitochondrial molecules and apoptosis of hepatocytes [21], thymocytes [19], and bursa of Fabricius cells provoked by AFB_1_ [27]. However, some death receptor molecules may not be related to AFB_1_-induced excessive cell death in the bursal cells [28], and the endoplasmic reticulum molecules may not be connected with the thymocyte apoptosis caused by AFB_1_ [19].

The spleen, as one of the peripheral immune organs, is the largest lymphoid organ of the body [29]. With a large number of T and B lymphocytes, it is the center of cellular and humoral immunity [30]. In peripheral lymphoid organs, apoptosis is linked with the proliferation and maturation of lymphocytes after antigen recognition [31]. However, the effect of death receptor and endoplasmic reticulum molecules on AFB_1_-induced splenic apoptosis remains practically unknown. Thus, we conducted this study in order to explore the alteration of death receptor and endoplasmic reticulum molecule expression in AFB_1_-induced apoptosis in splenocytes of chicken by histopathological observation, flow cytometry, immunohistochemistry and relative real-time fluorescent quantitative PCR (RT-qPCR) analysis. The outcomes from the present study could provide a reference for the further study of apoptosis mechanism caused by AFB_1_ in human and other animals in the future.

## RESULTS

### Growth performance

**Figure 1:**
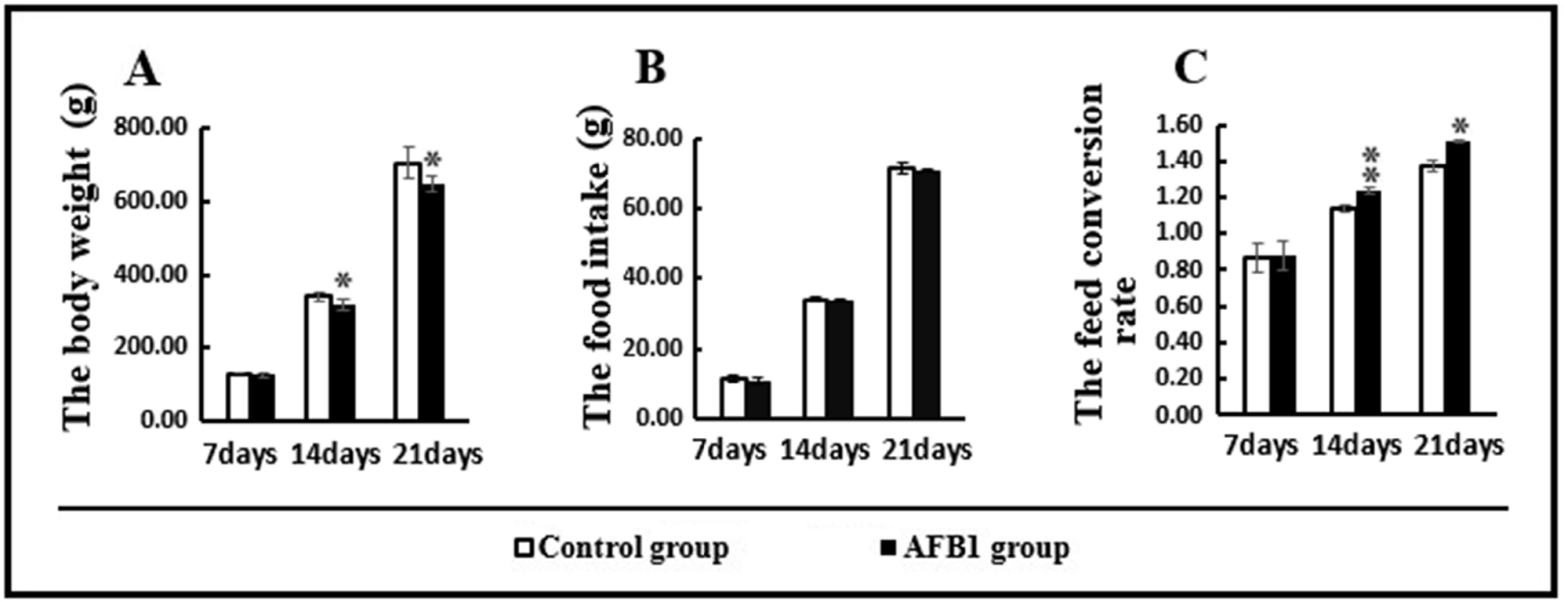
Growth performance (**A**) body weight; (**B**) food intake; (**C**) feed conversion rate. Data are presented with the means ± standard deviation, ^*^p < 0.05 and ^**^p < 0.01, compared with the control group.

The effects of dietary AFB1 on growth performance of chickens are shown in Figure 1. Compared with the control group, consumption of the AFB_1_ diet reduced body weight and caused poor feed conversion rate at 14 and 21 days (*p* < 0.05 or *p* < 0.01). Meanwhile, feed intake in the AFB_1_ group was not different from that in the control group (*p* > 0.05).

### Absolute weight and relative weight of spleen

**Figure 2:**
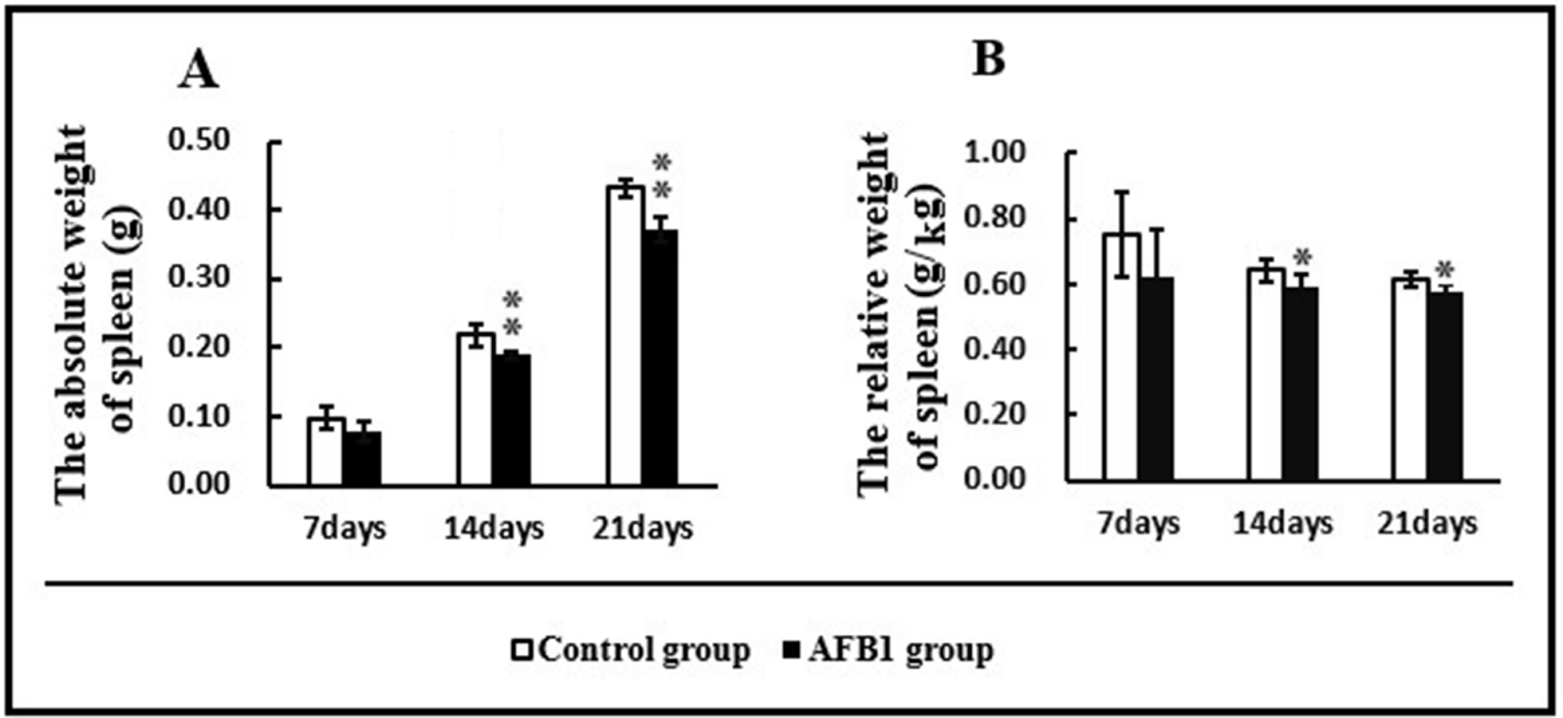
Absolute and relative weights of spleen (**A**) absolute weight of spleen; (**B**) relative weight of spleen. Data are presented with the means ± standard deviation (*n* = 6), ^*^p < 0.05 and ^**^p < 0.01, compared with the control group.

The absolute weight and relative weight of spleen in the AFB_1_ group were significantly lower than those in the control group on day 14 and 21 (*p* < 0.05 or *p* < 0.01) (Figure 2).

### Histopathological observation

**Figure 3:**
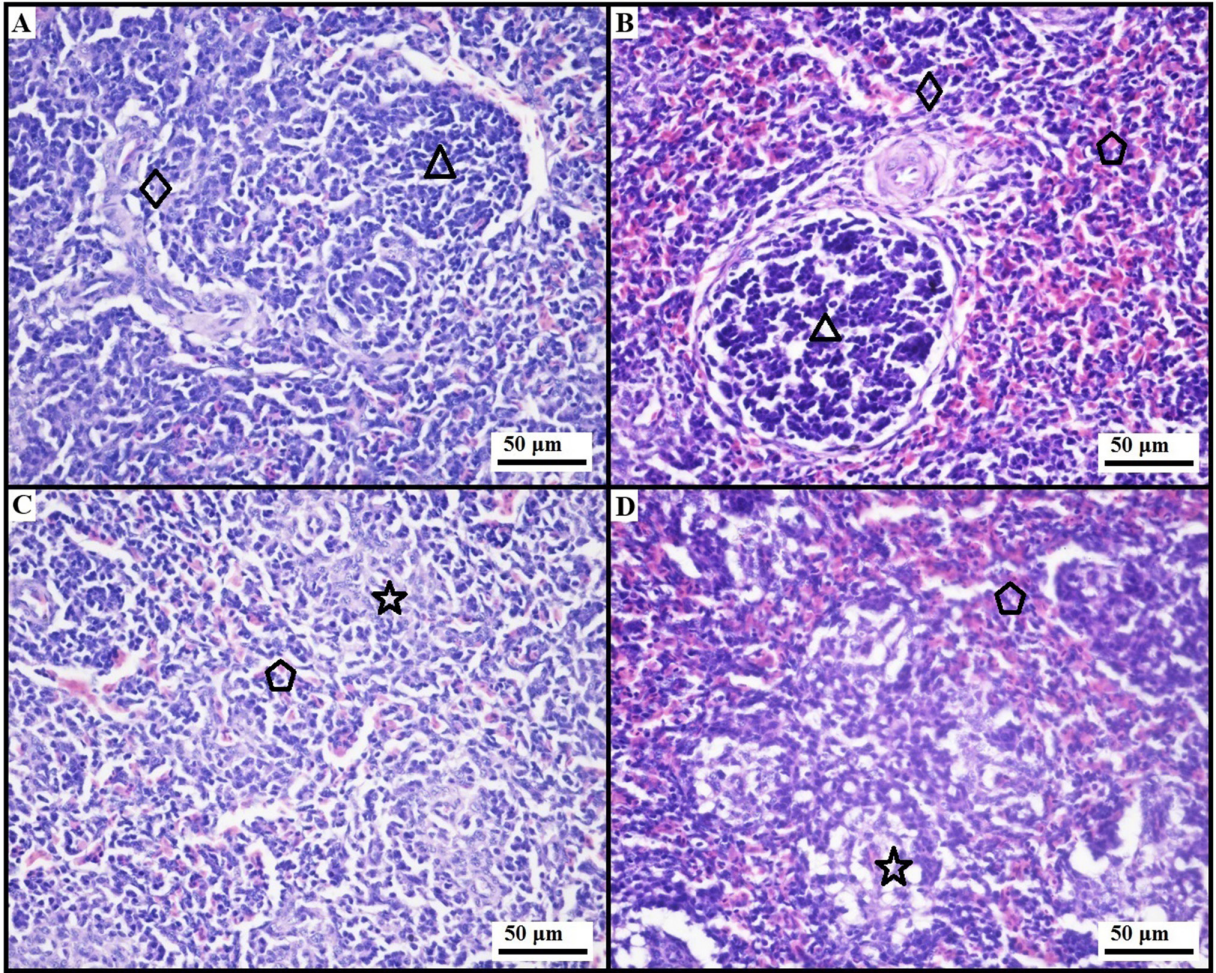
Histological observaton of the chicken spleen at 21 days of age (HE Staining, bar = 50 μm). Note: (**A**) the control group showing normal structure of periarterial lymphoid tissue (◇) and splenic nodule (△) ; (**B**) the AFB_1_ group showing slight congestion in the the red pulp (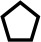) and lymphocyte depletion in the periarterial lymphoid tissue (◇) and splenic nodule (△); (**C**) the control group showing normal structure of periellipsoidal lymphoid tissue (☆) and red pulp (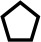); (**D**) the AFB_1_ group showing slight congestion in the red pulp (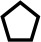) and the lymphocyte depletion in the periellipsoidal lymphoid tissue (☆).

The parenchyma of chicken spleen was classified as white and red pulps. The former was subdivided into the splenic nodule, periarterial lymphoid tissue, and periellipsoidal lymphoid tissue. Compared with mammals, the chicken spleen had indistinct red pulp and white pulp, rich periellipsoidal lymphoid tissue, and few splenic nodules. The slight congestion was seen in some places of the red pulp, and the lymphocyte density was chiefly decreased in the white pulp in the AFB_1_ group in comparison to the control group (Figure 3).

### Splenocyte apoptosis by TUNEL and flow cytometer analysis

**Figure 4:**
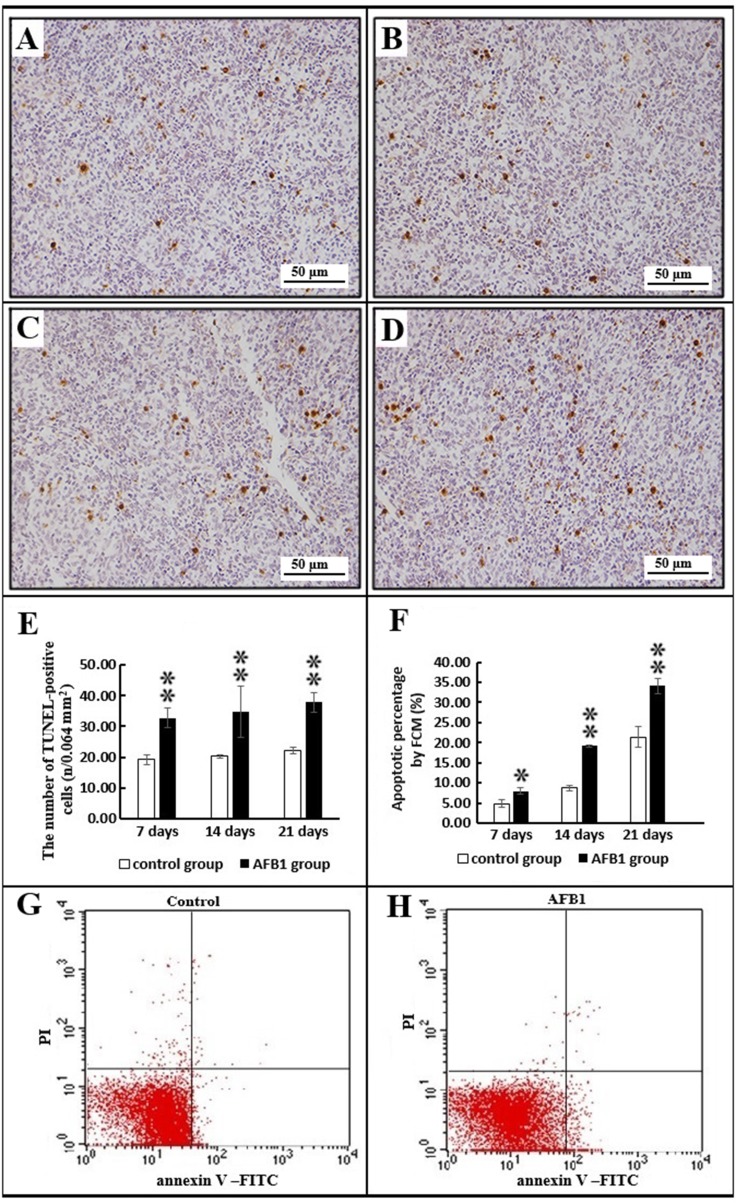
The splenocyte apoptosis by TUNEL immunohistochemistry and flow cytometry analysis Note: (**A–D**) TUNEL-positive cells in the control group (A) at 14 days, and AFB_1_ groups (B–D) at 7, 14 and 21 days of age (TUNEL assay, Scale bar: 50 μm). (**E**) The numbers of TUNEL-positive cells (microscopic quantitative analysis). Data are presented with the means ± standard deviation (*n* = 6). (**F**) Apoptotic percentages of splenocytes by flow cytometry assay. Data are presented with the means ± standard deviation (*n* = 6). (**G–H**) Scattergram of apoptotic splenocytes obtained by flow cytometry assay in the control group (G) and AFB_1_ group (H) at 21 days of age.

The nuclei of TUNEL-positive cells were stained brown. Under microscope, more positive cells in the AFB_1_ were observed than those in the control group during the experiment (Figure 4A–4D). Moreover, microscopic quantitative analysis also demonstrated the elevated number of positive cells in the AFB_1_ group (*p* < 0.01) when compared with the control group (Figure 4E). Flow cytometry assay showed the increased percentages of apoptotic splenocytes in the AFB_1_ group (*p* < 0.05 or *p* < 0.01) during the experiment (Figure 4F–4H).

### Expression levels of apoptosis associated genes by qRT-PCR

**Figure 5:**
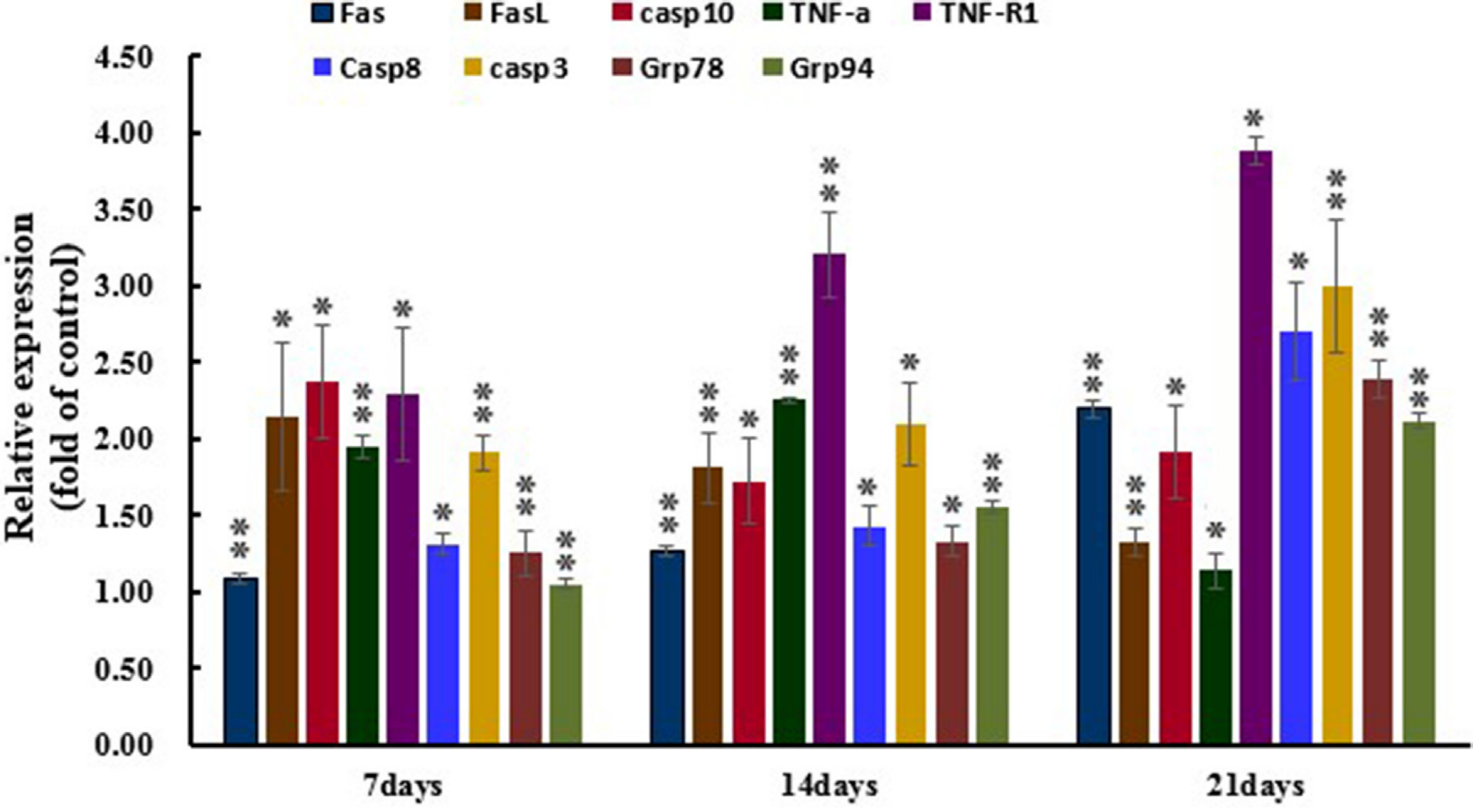
Expression levels of apoptosis associated gens by qRT-PCR Note: Data are presented with the means ± standard deviation (*n* = 6). ^*^P < 0.05, ^**^P < 0.01, compared with the control group.

The results of expression levels of apoptosis associated genes by qRT-PCR are shown in the Figure 5. Compared with the control group, the expression of Fas, FasL, TNF-α, TNF-R_1_, Caspase-10, Caspase-8, Caspase-3, Grp78 and Grp94 mRNA in the AFB_1_ group was significantly raised (*p* < 0.05 or *p* < 0.01).

## DISCUSSION

Our present study revealed that AFB_1_ significantly decreased body weight and affected feed conversion rate, suggesting that dietary AFB_1_ (0.6 mg/kg) affected the growth performance of the chicken, similar to previous reports [4].

As the the largest secondary immune organ, spleen takes part in activating the immune response to antigens, and in screening foreign substances [29]. The splenic size and gross morphology vary duo to different species and distension, but, the splenic weight is crucial in its functional evaluation [29]. The relative weight of spleen keeps relatively stable irrespective of age [29]. This study demonstrated that AFB_1_ could reduce the splenic absolute weight and relative weight along with lymphocytic depletion, similar to the report by Chen et al. [11], suggesting that this toxin had the detrimental effects on the development and immune function of spleen. Similarly, several reports also revealed that AFB_1_ could decrease the relative weights of central immune organs of chicken [8, 28, 32]. However, contrary reports also existed. For instance, Ortatatli et al. [33] reported that no statistical difference was found in the relative weight of spleen between the aflatoxins-treated broiler chicks and control ones. Peng et al. [34] demonstrated that aflatoxin-contaminated corn intake significantly increased the relative weight of chicken spleen. This discrepancy might partially be associated to the types of toxin because the aflatoxin-contamined diet in Ortatatli and Peng’s researches contained different kinds of mycotoxins including AFB_1_ [33, 34].

Apoptosis has an important role in deveolpment, differentiation, proliferation and homeostasis of cell, tissue and organ [35]. AFB_1_ directly or indirectly activated apoptotic process [36, 37], inducing apoptosis of several poultry and mammal cells [8, 9, 17, 19, 21–26]. The apoptotic cells could be evaluated by Flow cytometry and TUNEL assay [38, 39]. Our present research revealed an increased apoptosis in the AFB_1_ group demonstrated by TUNEL and flow cytometry, suggesting that AFB_1_ could lead excess apoptosis in the chickens’ splenocytes, in line with previous researches in thymocytes [19, 22], bursa of Fabricius cells [27] and renal cells [26]. Lymphocytes are the main components within the lymphoid organs, and severe lymphocyte depletion in the lymphoid organs was due to apoptosis [40]. Therefore, it is tempting to speculate that the increased apoptosis of splenocytes provoked by AFB_1_ might lead to the lymphocyte depletion, which may partly responsible for the declined splenic absolute and relative weights demonstrated in this study. Moreover, excessive apoptosis of lymphocytes was associated to immunosuppression in various circumstances [31]. Thus, our present results indicated that excessive apoptosis of spleen might cause immunosuppression in broilers exposed to AFB_1_. In addition, our present study revealed that the apoptotic percentage of the control group rose substantially by 21 days based on the flow cytometry assay, indicating that the normal apoptosis of the chicken’s spleen showed an increased changing pattern. Early researches also demonstrated that apoptsis of spleen was very obvious and presented development-related changes [41, 42]. Therefore, the present result that the apoptosis of AFB_1_-treated samples increased from 7 to 14 to 21 days may not be due to this increasing baseline, rather than from compounding effects of AFB_1_.

The signal pathways of apoptosis are complex and different under apoptosis induced factor stimulating. The death receptor and endoplasmic reticulum molecules are important molecules related to cell apoptosis, of which caspases are the the final executioners [43]. To provide a reference for the further study of apoptosis mechanism caused by AFB_1_, we explored the alteration of Fas, FasL, TNF-R_1_, Caspase-3, Caspase-8, Caspase-10 Grp94 and Grp78 expression in AFB_1_-induced apoptosis in splenocytes of chicken.

After bound with FasL or TNF-R_1_ and TNF-R_2,_ respectively, Fas and TNF-α were activated, then Caspase-10 and Caspase-8 were recruited and these Caspases including Caspase-3 were activated, leading the cell apoptosis [44–47]. The present study demonstrated that AFB_1_ diet led to the elevated expression of Fas, FasL, TNF-R_1_, Caspase-3, Caspase-8 and Caspase-10 mRNA expression in the spleen, which was consistent with earlier reports on the AFB_1_-induced apoptosis of thymocytes and hepatocytes [19, 21]. However, Yuan et al. reported that the mRNA expression of Fas, FasL, FADD, Caspase-8 and Caspase-10 in the bursa of Fabricius cells of the AFB_1_-treated chickens were not significant different from those of the control ones, suggesting that the excessive apoptosis of the bursal cells caused by AFB_1_ was not attributed to death receptor molecules [28]. Therefore, the effect of molecules involved in the AFB_1_-caused apoptosis was different and complicated, and may vary depending on different tissues.

The endoplasmic reticulum (ER) pathway is initiated by ER stress which causes the accumulation of misfolded or unfolded proteins in the ER [48, 49]. This accumulation activates the expression of Grp94 and Grp78 and enhance the protein folding machinery [49, 50]. If the unfolded protein reaction is unable to control the unfolded and misfolded protein levels in the ER, the apoptotic signaling provoked by ER is triggered by activating Caspase-12, and Caspase-3, and ultimately induces cell death [43, 49]. Our present result demonstrated that AFB_1_ could lead the elevated expression of Grp78 and Grp94 mRNA. This is consistent to previous report in the bursa of Fabricius cells [28]. However, contradictory result showed that the expression of Grp94 and Grp78 mRNA were not significant different between the AFB_1_-treated group and control group of thymocytes, suggesting that the ER molecules may not involve in the AFB_1_-induced apoptosis of thymocytes [19]. This is also confirmed that the effect of molecules related to the AFB_1_-induced apoptosis may be different duo to various cell types.

Our study demonstrated that dietary AFB_1_ (0.6 mg/kg) could cause the decline in the absolute weight and relative weight of chickens’ spleen along with mild congestion and lymphocytic depletion, and induce splenocyte apoptosis accompanied by the up-regulation of Fas, FasL, TNF-α, TNF-R_1_, Caspase-3, Caspase-8, Caspase-10, Grp78 and Grp94 mRNA expression.

## MATERIALS AND METHODS

### Animals and diets

The animal protocols used in this work and all procedures of the experiment were performed in compliance with the laws and guidelines of Sichuan Agricultural University Animal Care and Use Committee (Approval No: 2012–024). One hundred and forty four one-day-old healthy Cobb male chickens were purchased from a commercial rearing farm (Wenjiang poultry farm, Sichuan province, China), and randomly divided into two equal groups, namely control group (0 mg/kg AFB_1_) and AFB_1_ group (0.6 mg/kg AFB_1_). All of the chickens were put into cages with three replicates per group and 24 birds per replicate. The basal diet, namely the control diet, was formulated according to National Research Council (NRC, 1994) [51] and Chinese Feeding Standard of Chicken (NY/T33-2004). AFB_1_ was purchased from Sigma-Aldrich (USA, A6636). The AFB_1_-contaminated diet was made, similarly to the method described by Kaoud [52]. Briefly, 27 mg AFB_1_ farinose solid was dissolved into 30 mL methanol completely, and then the 30 mL mixture was mixed into 45 kg corn-soybean basal diet to formulate AFB_1_ diet of experimental groups containing 0.6mg/kg AFB_1_. The equivalent methanol was added into corn-soybean basal diet to formulate the control diet. Then the methanol of diets was evaporated at 98°F (37°C). The AFB_1_ concentrations were analyzed by HPLC (Waters, Milford, MA, USA) with fluorescence detection (Waters, Model 2475, Milford, MA, USA), and the AFB_1_ concentration was determined as < 0.001 mg/kg and 0.601 mg/kg in the control diet and AFB_1_-contaminated diet, respectively. Chickens were fed in cages with electrically heated units and provided with water as well as aforementioned diet *ad libitum* for 21 days.

### Growth performance

Body weight, feed intake, and feed conversion rate per cage were recorded weekly from 7 to 21 days of age.

### The absolute and relative weights of spleen

At 7, 14, and 21 days of age, six chickens in each group were randomly chosen, weighted, and humanely euthanized. And spleens were removed, and weighted. The relative weight of spleen was calculated by the following formula:

Relative weight of spleen = absolute weight of spleen (g)/body weight (kg)

### Histopathological observation

At 7, 14, and 21 days of age, the splenic tissue samples from six chickens in each group were collected and fixed in 4% paraformaldehyde (PFA), and then were dehydrated and embedded in paraffin wax. Blocks were cut into 5 µm sections with a microtome (Leica, Germany, RM2135) for haematoxylin and eosin (HE.) staining and TUNEL assay. The histological structure of the tissues was observed under light microscope and photographed with a digital camera (Nikon DS-Ri1, Japan).

### Apoptosis detection by TUNEL assay

TUNEL assay was performed with an apoptosis detection Kit (MK1020 Boster, Wuhan, China) according to the manufacturer’s instructions. Briefly, tissue sections were dewaxed with 100% xylene, and rehydrated in successive changes of 100%, 95%, 85% and 75% ethanol. After endogenous peroxidase activity was quenched for 10 min in 3% H_2_O_2_ with distilled water at 37°C, the sections were incubated with proteinase K diluted 1:200 in TBS at 37°C for 5–10 min in a humidified chamber. A labeling mixture containing digoxin-dUTP in TdT (Terminal deoxynucleotidyl Transferase) enzyme buffer was added to the sections and incubated at 37°C for 2 h. After three successive washings with TBS for 2 min, sections were covered with anti-digoxin-biotin conjugate diluted 1:100 in blocking regent and incubated for 30 min at 37°C. The tissues were then incubated for 1 h at 37°C with strept avidin-biotin-complex (SABC) diluted 1:100 in TBS. Labeling was visualized with 3′3′-diaminobenzidene. The sections were then counterstained with haematoxylin. For the negative control, representative sections were processed in the same way but incubation with TdT enzyme buffer was omitted.

The number of TUNEL-positive cells in the spleen was evaluated by Image-Pro Plus5.1 (USA) image analysis software. Briefly, photographs of TUNEL staining were taken with a digital microscope camera system (Nikon DS-Ri1, Japan). For each section, five fields of 0.064 mm^2^ (corresponding to five fields at 400 × magnification) were analyzed. By selecting “colour-chosen target” in the option bar of the morphologic analysis system, all TUNEL-positive cells in the field were marked in colour. Then, “calculating” in the option bar was selected to automatically calculate the number of TUNEL-positive cells. Results were expressed as the average of TUNEL-positive cells per 0.064 mm^2^ area.

### Apoptosis detection by flow cytometry

At 7, 14, and 21 days of the experiment, six chickens in each group were euthanized, and spleens were sampled to determine the percentage of apoptotic cells by flow cytometer, using the method by Chen et al. [53]. Briefly, the dissected spleens were thereupon homogenized to form a cell suspension and filtered, and then the cells were washed and resuspended in phosphate buffer at a concentration of 1 × 10^6^ cells/mL. 5 μL Annexin V-Fluorescein isothiocyanate (V-FITC) and 5 μL propidium iodide (PI) were added into 100 μL cell suspension, and incubated at 25°C for 15 min in the dark. 400 μL 1 × Annexin binding buffer was added to the mixture, and then the apoptotic cells were assayed by flow cytometer (BD FACSCalibur) within 1 h. The annexin V-FITC Kit was purchase from BD Pharmingen (USA, 556547).

### Expression levels of apoptotic regulator mRNAs by quantitative real-time PCR

Quantitative real-time PCR (qRT-PCR) assay was carried out as reported by Chen et al. [22]. Briefly, the spleens from six chickens in each groups at 7, 14, and 21 days of the experiment were obtained and stored in liquid nitrogen, respectively. Adding liquid nitrogen, the samples were crushed with pestle to homogenize until powdery, respectively. Total RNA was extracted from the powdery of samples using RNAiso Plus (9108/9109, Takara, Otsu, Japan). The mRNA was then reverse transcribed into cDNA using PrimScriptTM RT reagent Kit with gDNA Eraser (RR047A, Takara, Otsu, Japan). The cDNA was used as a template for qRT-PCR analysis.

For qRT-PCR reactions, 25 μL mixtures were made by using SYBR^®^ Premix Ex Taq™ II (DRR820A, Takara, Otsu, Japan), containing 12.5 μL Tli RNaseH Plus, 1.0 μL of forward and 1.0 μL of reverse primer, 8.5 μL RNAase-free water and 2 μL cDNA. Reaction conditions were set to 3 min at 95°C (first segment, one cycle), 10 s at 95°C and 30 s at Tm of a specific primer pair (second segment, 44 cycles) followed by 10 s at 95°C, and 72°C for 10 s (dissociation curve segment) using Thermal Cycler (C1000, BIO RAD, CA, USA). The mRNA expression of Fas, FasL, TNF-α, TNF-R_1_, Caspase-10, Caspase-8, Caspase-3, Grp78 and Grp94 was analyzed. β-actin was used as an internal control gene. Sequence of primers was obtained from GenBank of NCBI. Primers were designed with Primer 5, and synthesized by BGI Tech (Shenzhen, China) (Table 1). The qRT-PCR data were analyzed and fold change in expressions were calculated using the 2^-ΔΔCT^ method [54].

**Table 1:** Primer sequence for apoptotic genes

Gene symbol	Ref Seq	Forward primers	Reverse primers
Caspase-3	NM_204725	TGGCCCTCTTGAACTGAAAG	TCCACTGTCTGCTTCAATACC
Caspase-8	NM_204592	GTCTCCGTTCAGGTATCTGCT	TCTCAATGAAAACGTCCGGC
Caspase-10	XM_421936	CTGGGGGCTCCAAAAGTCC	AAAGGGGGACAAAGCCAACA
Fas	NM_001199487	TCCACCTGCTCCTCGTCATT	GTGCAGTGTGTGTGGGAACT
FasL	NM_001031559	GGCATTCAGTACCGTGACCA	CCGGAAGAGCACATTGGAGT
Grp78	NM_205491	GGTGTTGCTTGATGTGTGTCC	GCTGATTGTCAGAAGCTGTGG
Grp94	NM_204289	TGACCTGGATGCAAAGGTGGA	TTAAACCCCACACCATCCCTCAAC
TNF-α	AY765397	TCAGACCAGATGGGAAGGGA	ACTGGGCGGTCATAGAACAG
TNF-R1	NM_001030779	CCTGTCTGTCTTCCCTGTCC	GGTGCATGGGGTCTTTTCTA
β-actin	L08165	TGCTGTGTTCCCATCTATCG	TTGGTGACAATACCGTGTTCA

### Statistical analysis

The significance of difference between two groups was analyzed by variance analysis, and the results were expressed by mean ± standard deviation. The analyses were performed using the independent sample test of SPSS 20.0 software (IBM Corp, Armonk, NY, USA) for windows. Statistical significant differences were considered at *p* < 0.05 and markedly significant differences were considered at *p* < 0.01.

## References

[1] Liu Y, Wu F (2010). Global burden of aflatoxin-induced hepatocellular carcinoma: a risk assessment. Environ Health Persp.

[2] Bryden WL (2007). Mycotoxins in the food chain: human health implications. Asia Pac J Clin Nutr.

[3] Bennett JW (1999). Mycotoxins in agricultural and food safety. Mycopathologia.

[4] Yunus AW, Razzazi-Fazeli E, Bohm J (2011). Aflatoxin B_1_ in affecting broiler’s performance, immunity, and gastrointestinal tract: a review of history and contemporary issues. Toxins.

[5] Preisler V, Caspary WJ, Hoppe F, Hagen R, Stopper H (2000). Aflatoxin B_1_-induced mitotic recombination in L5178Y mouse lymphoma cells. Mutagenesis.

[6] Aguilar F, Harris CC, Sun T, Hollstein M, Cerutti P (1994). Geographic variation of p53 mutational profile in nonmalignant human liver. Science.

[7] Kalpana S, Aggarwal M, Srinivasa RG, Malik JK (2012). Effects of aflatoxin B_1_ on tissue residues of enrofloxacin and its metabolite ciprofloxacin in broiler chickens. Environ Toxicol Phar.

[8] Sur E, Celik I (2003). Effects of aflatoxin B_1_ on the development of the bursa of Fabricius and blood lymphocyte acid phosphatase of the chicken. Brit Poultry Sci.

[9] Sur E, Celik I (2005). Effects of aflatoxin B_1_ on the development of chicken thymus and blood lymphocyte alpha-naphthyl acetate esterase activity. Vlaams Diergen Tijds.

[10] Hinton DM, Myers MJ, Raybourne RA, Franckecarroll S, Sotomayor RE, Shaddock J, Warbritton A, Ming WC (2003). Immunotoxicity of aflatoxin B_1_ in rats: effects on lymphocytes and the inflammatory response in a chronic intermittent dosing study. Toxicol Sci.

[11] Chen K, Peng X, Fang J, Cui H, Zuo Z, Deng J, Chen Z, Geng Y, Lai W, Tang L (2014). Effects of dietary selenium on histopathological changes and T cells of spleen in broilers exposed to aflatoxin B_1_. Inter J Env Res Pub Heal.

[12] Celik I, Oguz H, Demet O, Donmez HH, Boydak M, Sur E (2000). Efficacy of polyvinylpolypyrrolidone in reducing the immunotoxicity of aflatoxin in growing broilers. Brit Poultry Sci.

[13] Potchinsky MB, Bloom SE (1993). Selective aflatoxin B1-induced sister chromatid exchanges and cytotoxicity in differentiating B and T lymphocytes *in vivo*. Environ Mol Mutagen.

[14] Okotieeboh GO, Kubena LF, Chinnah AD, Bailey CA (1997). Effects of beta-carotene and canthaxanthin on aflatoxicosis in broilers. Poultry Sci.

[15] Jiang M, Peng X, Fang J, Cui H, Yu Z, Chen Z (2015). Effects of aflatoxin b1 on T-cell subsets and mRNA expression of cytokines in the intestine of broilers. Int J Mol Sci.

[16] Manafi M, Murthy HNN, Swamy HDN (2012). Evaluation of different mycotoxin binders on aflatoxicosis in broiler breeders induced with aflatoxin B_1_: effects on biochemical and immunological parameters. American-Eurasian J Agric Environ Sci.

[17] Chen J, Chen K, Yuan S, Peng X, Fang J, Wang F, Cui H, Chen Z, Yuan J, Geng Y (2016). Effects of aflatoxin B_1_ on oxidative stress markers and apoptosis of spleens in broilers. Toxicol Ind Health.

[18] Peng X, Zhang K, Bai S, Ding X, Zeng Q, Yang J, Fang J, Chen K (2014). Histological lesions, cell cycle arrest, apoptosis and T cell subsets changes of spleen in chicken fed aflatoxin-contaminated corn. Inter J of Env Res Pub Heal.

[19] Peng X, Yu Z, Liang N, Chi X, Li X, Jiang M, Fang J, Cui H, Lai W, Zhou Y (2016). The mitochondrial and death receptor pathways involved in the thymocytes apoptosis induced by aflatoxin B_1_. Oncotarget.

[20] Cohen JJ, Duke RC, Fadok VA, Sellins KS (1992). Apoptosis and programmed cell death in immunity. Annu Rev Immunol.

[21] Mughal MJ, Peng X, Zhou Y, Fang J (2017). Aflatoxin B 1 invokes apoptosis via death receptor pathway in hepatocytes. Oncotarget.

[22] Chen K, Shu G, Peng X, Fang J, Cui H, Chen J, Wang F, Chen Z, Zuo Z, Deng J (2013). Protective role of sodium selenite on histopathological lesions, decreased T-cell subsets and increased apoptosis of thymus in broilers intoxicated with aflatoxin B_1_. Food Chem Toxicol.

[23] Yang X, Zhang Z, Wang X, Wang Y, Zhang X, Lu H, Wang SL (2013). Cytochrome P450 2A13 enhances the sensitivity of human bronchial epithelial cells to aflatoxin B_1_-induced DNA damage. Toxicol Appl Pharm.

[24] Peng X, Zhang S, Fang J, Cui H, Zuo Z, Deng J (2014). Protective roles of sodium selenite against aflatoxin B_1_-induced apoptosis of jejunum in broilers. Inter J Env Res Pub Heal.

[25] Raj HG, Kohli E, Rohil V, Dwarakanath BS, Parmar VS, Malik S, Adhikari JS, Tyagi YK, Goel S, Gupta K (2001). Acetoxy-4-methylcoumarins confer differential protection from aflatoxin B_1_-induced micronuclei and apoptosis in lung and bone marrow cells. Mutat Res/Genet Toxicol En.

[26] Yu Z, Wang F, Liang N, Wang C, Peng X, Fang J, Cui H, Jameel Mughal M, Lai W (2015). Effect of selenium supplementation on apoptosis and cell cycle blockage of renal cells in broilers fed a diet containing aflatoxin B_1_. Biol Trace Elem Res.

[27] Peng X, Chen K, Chen J, Fang J, Cui H, Zuo Z, Deng J, Chen Z, Geng Y, Lai W (2015). Aflatoxin B_1_ affects apoptosis and expression of Bax, Bcl-2, and Caspase-3 in thymus and bursa of Fabricius in broiler chickens. Environ Toxicol.

[28] Yuan S, Wu B, Yu Z, Fang J, Liang N, Zhou M, Huang C, Peng X (2016). The mitochondrial and endoplasmic reticulum pathways involved in the apoptosis of bursa of Fabricius cells in broilers exposed to dietary aflatoxin B_1_. Oncotarget.

[29] Cesta MF (2006). Normal structure, function, and histology of the spleen. Toxicol Pathol.

[30] Balogh P, Horváth G, Szakal AK (2004). Immunoarchitecture of distinct reticular fibroblastic domains in the white pulp of mouse spleen. J Histochem Cytochem.

[31] Rathmell JC, Thompson CB (2002). Pathways of apoptosis in lymphocyte development, homeostasis, and disease. Cell.

[32] Thaxton JP, Tung HT, Hamilton PB (1974). Immunosuppression in chickens by aflatoxin. Poultry Sci.

[33] Ortatatli M, Oğuz H, Hatipoğlu F, Karaman M (2005). Evaluation of pathological changes in broilers during chronic aflatoxin (50 and 100 ppb) and clinoptilolite exposure. Res Vet Sci.

[34] Peng X, Bai S, Ding X, Zeng Q, Zhang K, Fang J (2015). Pathological changes in the immune organs of broiler chickens fed on corn naturally contaminated with aflatoxins B_1_ and B_2_. Avian Pathol.

[35] Melnikova VI, Afanasyeva MA, Sapozhnikov AM, Zakharova LA (2006). Dynamics of apoptosis and proliferation in rat thymus and spleen during perinatal development (Ontogenesis). Russ J Dev Biol.

[36] Ribeiro DH, Ferreira FL, Da SV, Aquino S, Corrêa B (2010). Effects of aflatoxin B1 and fumonisin B_1_ on the viability and induction of apoptosis in rat primary hepatocytes. Int J Mol Sci.

[37] Brahmi D, Bouaziz C, Ayed Y, Mansour HB, Zourgui L, Bacha H (2011). Chemopreventive effect of cactus Opuntia ficus indica on oxidative stress and genotoxicity of aflatoxin B_1_. Nutr Metab.

[38] Vermes I, Haanen C, Steffensnakken H, Reutelingsperger C (1995). A novel assay for apoptosis flow cytometric detection of phosphatidylserine expression on early apoptotic cells using fluorescein labelled Annexin V. J Immunol Methods.

[39] Negoescu A, Guillermet C, Lorimier P, Brambilla E, Labat-Moleur F (1998). Importance of DNA fragmentation in apoptosis with regard to TUNEL specificity. Biomed Pharmacother.

[40] Solcan C, Solcan G, Oprisan B, Spataru M, Spataru C, Floristean V (2014). Immunotoxic action of aflatoxin B1 against lymphoid organs is coupled with the high expression of Bcl-2 by reticuloepithelial cells in broiler chickens. J Anim Vet Adv.

[41] Mel’nikova VI, Afanas’eva MA, Sapozhnikov AM, Zakharova LA (2006). Dynamics of apoptosis and proliferation in the rat thymus and spleen during perinatal development. Russ J Dev Biol.

[42] Itzhaki O, Skutelsky E, Kaptzan T, Sinai J, Michowitz M, Huszar M, Leibovici J (2003). Ageing-apoptosis relation in murine spleen. Mech Ageing Dev.

[43] Wang H, Liu H, Zheng ZM, Zhang KB, Wang TP, Sribastav SS, Liu WS, Liu T (2011). Role of death receptor, mitochondrial and endoplasmic reticulum pathways in different stages of degenerative human lumbar disc. Apoptosis.

[44] Park JB, Kim KW, Han CW, Chang H (2001). Expression of Fas receptor on disc cells in herniated lumbar disc tissue. Spine.

[45] Schulze-Osthoff K, Ferrari D, Los M, Wesselborg S, Peter ME (2013). Apoptosis signaling by death receptors. Eur J Biochem.

[46] Park JB, Chang H, Kim KW (2001). Expression of Fas ligand and apoptosis of disc cells in herniated lumbar disc tissue. Spine.

[47] Wallach D (1996). Suicide by order: some open questions about the cell-killing activities of the TNF ligand and receptor families. Cytokine Growth F R.

[48] Ron D, Walter P (2007). Signal integration in the endoplasmic reticulum unfolded protein response. Nat Rev Mol Cell Bio.

[49] Du L, He F, Kuang L, Tang W, Li Y, Chen D (2017). eNOS/iNOS and endoplasmic reticulum stress-induced apoptosis in the placentas of patients with preeclampsia. J Hum Hypertens.

[50] Malhotra JD, Kaufman RJ (2007). The endoplasmic reticulum and the unfolded protein response. Semin Cell Dev Biol.

[51] Dale N (1994). National research council nutrient requirements of poultry-ninth revised edition (1994). J Appl Poultry Res.

[52] Kaoud HA (2012). Innovative methods for the amelioration of aflatoxin (AFB_1_) effect in broiler chicks. Sci J App Res.

[53] Chen T, Cui H, Cui Y, Bai C, Gong T, Peng X (2011). Cell-cycle blockage associated with increased apoptotic cells in the thymus of chickens fed on diets high in fluorine. Hum Exp Toxicol.

[54] Livak KJ, Schmittgen TD (2001). Analysis of relative gene expression data using real-time quantitative PCR and the 2^−ΔΔCT^ method. Methods.

